# Association between exposure to different sources of advertising and the use of conventional cigarettes in Peruvian adolescents, 2019

**DOI:** 10.18332/tid/162326

**Published:** 2023-05-13

**Authors:** María Lucero Pareja Llerena, Akram Hernández-Vásquez, Gianfranco W. Basualdo-Meléndez, Diego Azañedo

**Affiliations:** 1 Facultad de Ciencias de la Salud, Universidad Científica del Sur, Lima, Peru; 2 Centro de Excelencia en Investigaciones Económicas y Sociales en Salud, Vicerrectorado de Investigación, Universidad San Ignacio de Loyola, Lima, Peru

**Keywords:** surveys and questionnaires, tobacco smoking, advertising, adolescent, Peru

## Abstract

**INTRODUCTION:**

The objective of this study was to evaluate the association between exposure to different sources of tobacco advertising and the consumption of conventional cigarettes in Peruvian adolescents.

**METHODS:**

This was a cross-sectional analytical study of secondary data from the Global Youth Tobacco Survey (GYTS) 2019 in Peru. The population consisted of adolescents aged 13–15 years. Generalized Linear Poisson family models were used to estimate prevalence ratios with their 95% confidence intervals (95% CI), which measured the strength of the association between exposure to advertising sources and conventional cigarette consumption.

**RESULTS:**

Data from 2083 adolescents who provided information on exposure to television advertising, 1092 on outdoor advertising, and 2008 about online advertising were analyzed. A higher probability of conventional cigarette consumption was noted in those exposed to ads via television (aPR=1.85; 95% CI: 1.28–2.69; p=0.002) and online (aPR=1.90; 95% CI: 1.40–2.58; p<0.001), in comparison with those not exposed.

**CONCLUSIONS:**

Tobacco advertising, promotion and sponsorship (TAPS) through television and online media are significantly associated with an increase in the consumption of conventional cigarettes among adolescents aged 13–15 years. Therefore, it is necessary to implement comprehensive bans on TAPS in Peru focused on these media to prevent the tobacco industry from continuing to introduce advertising to encourage tobacco consumption.

## INTRODUCTION

Around the world, smoking represents the main cause of preventable death, with over 8.7 million deaths per year, and it is also the cause of tens of millions of preventable, mainly chronic, diseases^1^. In 2022, the Centers for Disease Control and Prevention (CDC) reported that nearly 2 in 100 high school students in the US (2.0%) had smoked conventional cigarettes in the past 30 days^2^. In Peru, in 2014, the World Health Organization (WHO) and the CDC conducted the Global Youth Tobacco Survey (GYTS), estimating that 9% of adolescents aged 13–15 years smoked tobacco (males, 10.5%; females, 7.4%)^3^. The survey was repeated in 2019 and showed that 6.4% of the same population group smoked tobacco (males, 7.1%; females, 5.6%)^4^. However, despite the decrease in the prevalence, this figure is concerning since tobacco use can increase the risk of nicotine addiction, reduced lung function, reduced lung growth, and early cardiovascular damage^5^.

In view of this problem, the WHO implemented the MPOWER strategy for the control of tobacco consumption, with one of its components being the enforcement of bans on tobacco advertising, promotion and sponsorship (TAPS)^1^. Despite the potential effectiveness of this measure in reducing tobacco use and the incidence of new smokers, by 2020, the WHO reported that only 57 countries complied with comprehensive TAPS bans^1^. Additionally, in 2021, the WHO and a study carried out in Latin American countries reported that Peru had a weak and limited strategy due to the absence of a complete comprehensive law for the prohibition of TAPS^6,7^.

Previous international studies have found a relationship between advertising and tobacco use in adolescents. A 2011 Cochrane meta-analysis evaluated 19 longitudinal studies, mainly from the US, in which 18 studies reported that non-smoking adolescents exposed to TAPS were more likely to become smokers^8^. Another study in adolescents from South America that used the GYTS databases from 1999 to 2008 found a positive association between exposure to tobacco advertising (television, billboards, and events) and experimentation with conventional cigarettes in non-smokers and an increased intensity of habitual consumption in smokers^9^. On the other hand, in 2019, the tobacco industry in the US invested approximately 22.5 million dollars per day in the promotion of conventional cigarettes, which was demonstrated to be effective in influencing adolescents to initiate tobacco consumption^8,9^. For this reason and given that adolescents are part of the target audience of the tobacco industry, evidence is required to strengthen decision-making on anti-smoking public health strategies, mainly aimed at reducing exposure to advertising on its consumption.

Indeed, while the impact of TAPS is known, the evidence used for its analysis is not recent, and advertising on tobacco consumption has currently been diversified through different media to which adolescents have access. Therefore, each of these media’s contribution to the use of cigarettes in this population requires evaluation. This can be achieved with the latest data from the GYTS 2019 in Peru, which provides information on tobacco use in adolescents. Likewise, this survey explores different sources of advertising (television, online and outdoor) individually and adolescents’ tobacco consumption. Taking all the above into account, the objective of this study was to evaluate the association between exposure to different sources of tobacco advertising and the consumption of conventional cigarettes in Peruvian adolescents.

## METHODS

### Study design and data sources

An analytical cross-sectional study was conducted using secondary data from the most recent (2019) GYTS conducted in Peru by the National Center for Epidemiology, Prevention and Disease Control of the Peruvian Ministry of Health.

The GYTS is a standardized, internationally comparable, school-based survey performed on adolescents aged 13–15 years worldwide. The GYTS is part of the Global Tobacco Surveillance System that helps countries monitor tobacco control activities under the WHO Framework Convention on Tobacco Control and is funded by the WHO and the CDC^10^. In Peru, GYTS 2019 used a standardized two-stage sampling methodology that provides a nationally representative sample. The first stage consisted of selecting schools with a probability of being selected proportionately to enrolled students. The second stage consisted of randomly selecting classrooms in each school selected. All students in the selected classrooms were eligible to complete an anonymous, standardized, self-administered questionnaire voluntarily. A detailed description of the 2019 GYTS is available on the website of the US CDC and the WHO^11,12^. The 2019 GYTS databases are publicly accessible on the CDC website: https://www.cdc.gov/tobacco/global/gtss/gtssdata/index.html.

### Participants

The 2019 GYTS included all high school students in the selected classrooms and excluded those who did not consent or did not attend school the day of questionnaire administration. An overall response rate of 89% and a population of 4148 students were obtained^4^.

**Figure 1:**
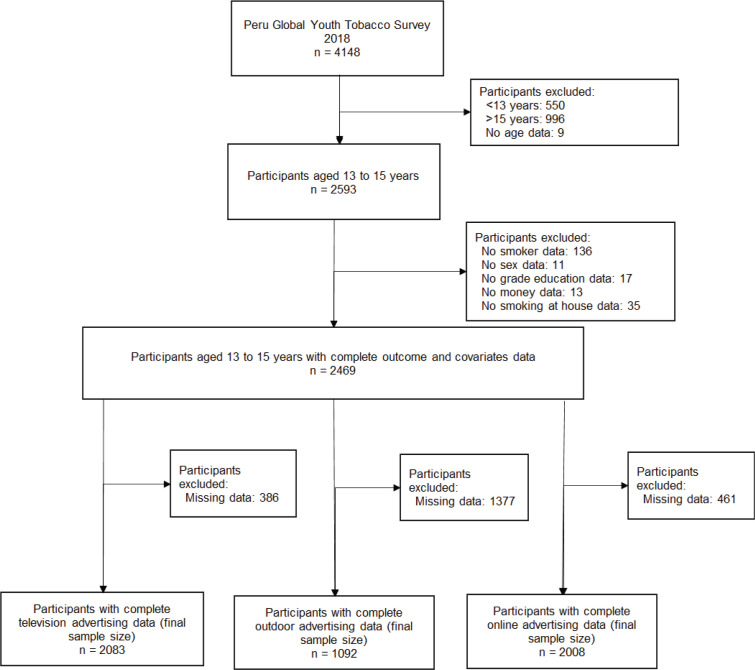
Flowchart of the selection of participants included in the study

The present study included students aged 13–15 years with data for the variables of interest. It was decided to include this age group because it represents the target population of the GYTS^4^. The final sample for the analysis was composed of 2083 schoolchildren with information on exposure to television advertising, 1092 schoolchildren with information on exposure to outdoor advertising and 2008 schoolchildren with information on exposure to online advertising. The flowchart of participant inclusion is presented in Figure 1.

### Variables and measures

*Outcome*

Current use/consumption of conventional cigarettes was defined as students aged 13–15 years who reported having consumed cigarettes at least one day during the last 30 days through the question: ‘During the last 30 days, how many days did you smoke cigarettes?’.

### Exposures

Exposure to the different sources of advertising was divided into three types and the following survey questions were considered for the construction of this variable:

*Television advertising*

This was a categorial variable with a dichotomous response (yes/no) that was constructed from an affirmative response to at least one of the following questions:

- ‘During the past 30 days, did you see any people using tobacco on TV, in videos, or in movies?’; and
- ‘During the past 30 days, did you see any tobacco product brand names when you watched sports events or any other programs on TV?’.

*Outdoor advertising*

This was a categorial variable with a dichotomous response (yes/no) that was constructed from an affirmative response to at least one of the following questions:

- ‘During the past 30 days, did you see any advertisements or promotions for tobacco products at points of sale such as stores, supermarket, kiosks or shops?’;
- ‘During the past 30 days, did you see any advertisements or promotions for tobacco products at sports events, fairs, concerts, or community events?’; and
- ‘During the past 30 days, did you see any advertisements for tobacco products on the beaches?’.

*Online advertising*

This was a categorial variable with a dichotomous response (yes/no) that was constructed from an affirmative response to at least one of the following questions:

- ‘During the past 30 days, did you see any advertisements for tobacco products on the Internet or social network (Facebook, YouTube, Snapchat)?’; and
- ‘During the past 30 days, did you see any videos on the Internet or social network that promote smoking tobacco or make smoking tobacco look fun/cool?’.

### Covariates

The following variables were included to control for possible confounding effects according to epidemiological criteria: sex (male/female); age (13, 14, and 15 years); Exposure to cigarrete smoking at home in the last 7 days (no/yes), constructed from the question: ‘During the past 7 days, on how many days has anyone smoked inside your home, in your presence?’; grade of secondary education (1st, 2nd, 3rd, 4th, and 5th); and money available per week to spend (in Peruvian Soles, with an exchange rate ranging between 3.30 and 3.35 PEN for US$1, in 2019) was measured with the question: ‘During an average week, how much money do you have that you can spend on yourself, however you want?’. Response choices were: ‘do not have’, <5, 5–9, 10–20, 21–40, 41–50, and >50 PEN.

### Data management and statistical analysis

The GYTS 2019 database in Microsoft Access format was downloaded and preprocessed using R programming language (version 4.0.1, R Foundation for Statistical Computing, Vienna, Austria). Stata 17.0 (StataCorp LP, Texas, USA) was used to perform all the statistical analyses. A descriptive analysis of the characteristics of the participants was performed according to the exposure assessed. Chi-squared tests of independence with Rao-Scott correction were applied to assess the bivariate association between the covariates of interest and cigarette smoking and advertising sources^13^. In addition, Poisson family generalized linear models with log link function were used to perform the crude and adjusted analyses to estimate prevalence ratios (PR) along with their 95% confidence intervals (95% CI), which measured the strength of the association between exposure to advertising sources and conventional cigarette consumption, after controlling for potential confounders (sex, age, ‘Exposure to cigarrete smoking at home in the last 7 days’, grade, and money available to spend per week) in the adjusted analysis. The selection of these variables followed an epidemiological criterion (common ancestors between the exposures and outcome evaluated). Sampling weights, strata, and primary sampling units contained in the database were included in all the analyses using the svy command of Stata 17.0 statistical software (StataCorp LP, Texas, USA). The statistical analysis was conducted using two-tailed tests, and a p<0.05 was used to define statistical significance.

### Ethical considerations

The Institutional Research Ethics Committee of the Universidad Científica del Sur approved this study and written informed consent was obtained from the parents/guardians of the students who participated in the survey. The anonymized databases of the GYTS of Peru and other countries are freely available on the CDC web page: https://www.cdc.gov/tobacco/global/gtss/gtssdata/index.html.

## RESULTS

Among the participants with information on exposure to television advertising on tobacco use, the majority were female (50.9%) and aged 13 years (35.8%). As for those with information regarding outdoor advertising, the majority were male (51.6%) and aged 13 years (37.5%). Likewise, a higher proportion of male participants (50.5%) aged 14 years (35.7%) presented information regarding online advertising. In addition, most of the students in the different groups of advertising exposure (television, outdoor, and online) were in the second year of high school (34.1%, 35.1% and 32.3%, respectively) and had <5 PEN available per week for their own expenses (27.6%, 29.9% and 27.4%, respectively). Likewise, 10.7%, 9.0% and 10.2% of the respondents in the advertising exposure groups stated that in the last seven days, somebody had smoked at least one day in their presence at home. Similarly, 4.4%, 4.8% and 4.5%, respectively, reported being smokers (Table 1).

**Table 1:** Characteristics of the Peruvian adolescents participating in the GYTS 2019 included in the study

*Characteristics*	*Sample for television advertising (N=2083)*	*Sample for outdoor advertising (N=1092)*	*Sample for online advertising (N=2008)*
	n (%)*	n (%)*	n (%)*
**Sex**			
Male	990 (49.1)	543 (51.6)	972 (50.5)
Female	1093 (50.9)	549 (48.4)	1036 (49.5)
**Age** (years)			
13	753 (35.8)	411 (37.5)	704 (34.4)
14	725 (35.4)	390 (35.4)	703 (35.7)
15	605 (28.8)	291 (27.1)	601 (29.9)
**Exposure to cigarrete smoking at home in the last 7 days**			
No	1861 (89.3)	992 (91.0)	1793 (89.8)
Yes	222 (10.7)	100 (9.0)	215 (10.2)
**Grade of secondary education**			
1st	311 (14.9)	185 (16.2)	297 (14.7)
2nd	695 (34.1)	373 (35.1)	648 (32.3)
3rd	609 (28.9)	328 (29.3)	599 (30.2)
4th	417 (19.1)	178 (16.1)	413 (19.7)
5th	51 (2.9)	28 (3.2)	51 (3.1)
**Money available per week to spend** (PEN)			
None	274 (14.1)	141 (13.0)	264 (13.5)
<5	568 (27.6)	321 (29.9)	538 (27.4)
5–9	485 (23.7)	251 (23.4)	455 (23.3)
10–20	475 (22.1)	225 (19.8)	450 (21.8)
21–40	142 (6.4)	76 (7.2)	150 (6.9)
41–50	60 (3.0)	36 (3.5)	68 (3.7)
>50	79 (3.1)	42 (3.2)	83 (3.4)
**Current use of conventional cigarettes**			
No	1986 (95.6)	1036 (95.2)	1913 (95.5)
Yes	97 (4.4)	56 (4.8)	95 (4.5)

* Percentages include the weights and sample specifications of the Peruvian GYTS 2019. PEN: Peruvian Soles (exchange rate ranging between 3.30 and 3.35 PEN for US$1, in 2019).

In the bivariate analysis, only the variable ‘money available for own expenses’ was significantly associated with the different types of exposure (p=0.007, p=0.008, and p=0.006, respectively). On the other hand, the variable ‘exposure to cigarette smoking at home in the last 7 days’ was significantly associated with television exposure (p=0.025) and online exposure (p<0.001), presenting a marginally significant association (p=0.053) with outdoor exposure (Table 2). On the other hand, Table 3 shows that the variable ‘exposure to cigarettes at home in the last 7 days’ was associated with cigarette consumption in the study population with information from television (p<0.001), outdoor (p<0.001), and online exposure (p<0.001), while the association with the outcome was only marginally significant for the variable ‘money available for own expenses’ in the different exposure groups.

**Table 2:** Characteristics of the Peruvian adolescents participating in the GYTS 2019 included in the study according to their exposure to different advertising sources

*Characteristics*	*Exposure to television advertising*			*Exposure to outdoor advertising*			*Exposure to online advertising*		
	*No (N=1525)*	*Yes (N=558)*		*No (N=1021)*	*Yes (N=71)*		*No (N=1722)*	*Yes (N=286)*	
	*%*	*%*	p*	*%*	*%*	p*	*%*	*%*	p*
**Sex**									
Male	49.6	47.9	0.573	51.4	54.2	0.697	50.8	48.8	0.608
Female	50.4	52.1		48.6	45.8		49.2	51.2	
**Age** (years)									
13	36.9	32.8	0.354	37.5	37.9	0.803	34.5	33.6	0.655
14	34.7	37.4		35.6	32.5		35.3	38.1	
15	28.4	29.9		26.9	30.1		30.2	28.3	
**Exposure to cigarrete smoking at home in the last 7 days**									
No	91.5	83.5	0.025	91.7	81.1	0.053	91.9	76.9	<0.001
Yes	8.5	16.5		8.3	18.9		8.1	23.1	
**Grade of secondary education**									
1st	15.0	14.5	0.947	16.0	19.3	0.689	14.9	13.9	0.513
2nd	34.1	34.2		34.8	40.7		31.6	36.7	
3rd	29.0	28.7		29.8	21.5		30.8	26.0	
4th	18.8	20.1		16.1	16.9		19.6	20.8	
5th	3.1	2.5		3.3	1.6		3.1	2.6	
**Money available per week to spend** (PEN)									
None	13.5	15.6	0.007	12.2	24.8	0.008	14.0	10.4	0.006
<5	28.6	24.8		30.6	20.2		28.1	23.0	
5–9	23.5	24.2		24.0	15.6		24.1	18.1	
10–20	23.4	18.6		19.8	20.4		21.0	26.7	
21–40	5.9	7.9		6.9	10.7		6.4	9.9	
41–50	2.4	4.8		3.7	0.7		3.3	5.8	
>50	2.7	4.0		2.9	7.5		3.0	5.9	

Estimates include the weights and sample specifications of the Peruvian GYTS 2019. PEN: Peruvian Soles (exchange rate ranging between 3.30 and 3.35 PEN for US$1, in 2019).

* Rao-Scott chi-squared test.

**Table 3:** Characteristics of the Peruvian adolescents participating in the GYTS 2019 included in the study according to the current use of conventional cigarettes

*Characteristics*	*Sample for television advertising*			*Sample for outdoor advertising*			*Sample for online advertising*		
	*Current use of conventional cigarretes*			*Current use of conventional cigarretes*			*Current use of conventional cigarretes*		
	*No (N=1986) %*	*Yes (N=97) %*	p*	*No (N=1036) %*	*Yes (N=56) %*	p*	*No (N=1913) %*	*Yes (N=95) %*	p*
**Sex**									
Male	49.0	52.5	0.551	51.6	51.5	0.989	50.4	52.5	0.739
Female	51.0	47.5		48.4	48.5		49.6	47.5	
**Age** (years)									
13	36.2	27.5	0.276	38.0	28.4	0.427	34.8	25.7	0.236
14	35.3	38.3		35.2	38.8		35.7	36.1	
15	28.5	34.2		26.8	32.8		29.5	38.2	
**Exposure to cigarrete smoking at home in the last 7 days**									
No	90.2	68.9	<0.001	92.5	62.0	<0.001	90.8	67.9	<0.001
Yes	9.8	31.1		7.5	38.0		9.2	32.1	
**Grade of secondary education**									
1st	15.0	11.0	0.752	16.2	17.3	0.748	14.8	13.3	0.896
2nd	33.9	38.8		34.7	43.4		32.1	35.6	
3rd	29.0	27.3		29.6	22.7		30.1	30.9	
4th	19.1	20.8		16.2	14.4		19.8	18.2	
5th	3.0	2.0		3.2	2.2		3.1	1.9	
**Money available per week to spend** (PEN)									
None	14.2	11.4	0.069	12.7	18.4	0.055	13.5	12.8	0.080
<5	28.1	17.2		30.5	17.7		27.7	20.5	
5–9	23.7	23.4		23.9	14.6		23.5	17.5	
10–20	22.0	23.3		19.8	20.1		21.8	22.2	
21–40	6.2	11.0		7.0	11.0		6.8	10.2	
41–50	2.8	7.2		3.2	8.7		3.4	9.4	
>50	2.9	6.4		2.9	9.4		3.2	7.3	

Estimates include the weights and sample specifications of the Peruvian GYTS 2019. PEN: Peruvian Soles (exchange rate ranging between 3.30 and 3.35 PEN for US$1, in 2019).

* Rao-Scott chi-squared test.

In the crude analysis, all three types of exposure to sources of tobacco advertising significantly increased the probability of cigarette smoking. The results adjusted for confounding variables showed that adolescents with television exposure to tobacco advertising had 1.85 times (95% CI: 1.28–2.69) higher smoking prevalence than those without television exposure (p=0.002). On the other hand, adolescents with online exposure to tobacco consumption advertising had a prevalence of smoking 1.90 times (95% CI: 1.40–2.58) greater than those without this type of exposure (p<0.001). Finally, exposure to outdoor advertising was not significantly associated with cigarette consumption (aPR=1.57; 95% CI: 0.75–3.28; p=0.222) (Table 4).

**Table 4:** Association between exposure to a source of advertising and current use of conventional cigarettes in Peruvian adolescents participating in the GYTS 2019 included in the study

*Characteristics*	*Crude models*		Adjusted models*	
	*PR (95% CI)*	*p*	*aPR (95% CI)*	*p*
**Exposure to television advertising** (n=2083)				
No (Ref.)	1		1	
Yes	2.34 (1.48–3.71)	0.001	1.85 (1.28–2.69)	0.002
**Exposure to outdoor advertising** (n=1092)				
No (Ref.)	1		1	
Yes	2.60 (1.09–6.21)	0.033	1.57 (0.75–3.28)	0.222
**Exposure to online advertising** (n=2008)				
No (Ref.)	1		1	
Yes	2.82 (1.99–3.99)	<0.001	1.90 (1.40–2.58)	<0.001

* Adjusted models; adjusted for sex, age, exposure to cigarrete smoking at home in the last 7 days, grade, and money available to spend per week (PEN). Estimates include the weights and sample specifications of the Peruvian GYTS 2019. PR: prevalence ratio. aPR: adjusted prevalence ratio.

## DISCUSSION

The objective of this study was to evaluate the association between exposure to different sources of advertising on tobacco consumption and the consumption of conventional cigarettes in Peruvian adolescents. Three subsamples of adolescents aged 13–15 years evaluated in the GYTS of Peru were analyzed, with 26.8% being exposed to television advertising, 6.5% to outdoor advertising, and 14.2% to online advertising. Additionally, between 4.4% and 4.8% of the adolescents participating in the different subsamples of the study stated that they were smokers. Exposure to television and online advertising was associated with an 85% and 90% higher probability of conventional cigarette consumption in the study population, respectively. Due to the negative consequences of cigarette use, especially in early life, it is necessary for Peru to implement comprehensive bans on TAPS, with an emphasis on the adolescent population, to avoid the incidence of new users.

The results obtained in this study show that exposure to television advertising was associated with an 85% higher probability of smoking conventional cigarettes. This is consistent with previous studies conducted in Latin America and the US^9,14^. In this regard, when the control measures on tobacco advertising are partial, tobacco companies exploit legal loopholes or use unregulated types of advertising, and thus, the strategies have little to no effect on tobacco consumption^1^. For example, although there are strategies to improve the monitoring and regulation of tobacco on television in the Middle East, the tobacco industry promotes its use in movies and seeks their transmission during times with greater number of viewers, such as during Ramadan^15^. According to the 2021 WHO report, in Peru, there is full compliance with a restriction at the level of direct advertising on national and international television^6^. This measure was first applied in 2006 and is stipulated by Law No. 28705, Chapter IV, Article 17^16^. However, the same WHO report in Peru describes the lack of measures at the indirect advertising level, such as including tobacco products or brands on television or in movies. Furthermore, the report notes that the prohibition law does not explicitly address cross-border advertising^6^. Therefore, it is possibly due to these factors that in 2019, almost 3 in 10 adolescents in Peru were still exposed to television advertising despite the prohibitions.

No significant association was found between outdoor advertising exposure and conventional cigarette consumption. On the contrary, when using data from the GYTS in South America from 1999 to 2008, it was determined that exposure to advertising during events, such as concerts, fairs, and sporting events, was associated with regular consumption of conventional cigarettes; however, exposure to advertising posters had no association^9^. The present study considered exposure to advertising posters as independent of the exposure to advertising in events. In this regard, it is likely that much of the advertising in events is shown on advertising posters, which we considered could not be evaluated separately. Furthermore, another factor which may influence the absence of an identified association may be due to the fact that the questionnaire was administered in classrooms during school periods, during which adolescents’ access to beaches or events, such as sporting events, fairs, or concerts may be limited, and they are not in contact with outdoor advertising. Furthermore, despite the ban on outdoor advertising being part of the strategy to reduce tobacco consumption proposed by the WHO, in Peru there are still no regulations for outdoor advertising, such as billboards or advertising at points of sale, and the only restriction stipulated by the law is the prohibition of sponsorship of events aimed at minors^6^. Nevertheless, even in certain countries where the corresponding legislation is in effect, the use of outdoor tobacco advertising continues to be observed, as occurred during the 2020 Indian Premier League during which it was observed that despite having comprehensive bans, large tobacco companies were the main sponsors of two participating teams in direct violation of the tobacco control law in India^17^.

The advertising medium with which adolescents interact most frequently today is online advertising^18^. Each website, social network or application has advertising within its platform, which does not always have regulations for underage users, thereby allowing them to come in contact with TAPS. The findings of the present study show that exposure to online advertising was associated with a 90% greater probability of smoking conventional cigarettes, being consistent with previous literature^18,1^. In this regard, in Peru there is no comprehensive ban at the online level; it only establishes that ads directly promoting tobacco products are not allowed, but online sales continue^6^. Considering the wide accessibility and ease that the Internet provides, and the important association of TAPS online with the consumption of conventional cigarettes, greater importance should be given to the management of online tobacco advertisements.

Previous studies have used different ways of grouping the media, with some adopting the general term advertising while others focus on analyzing a subtype of advertising^9,15,17–19^. In contrast, our study grouped the questions and classified them into television, outdoor and online advertising, which may make the comparisons of results difficult. However, this provided the advantage of discriminating which types of advertising have a greater association with the consumption of conventional cigarettes, thus allowing a better analysis and usefulness of the results in targeting strategies to reduce tobacco consumption in Peruvian adolescents.

In 2021, the WHO reported 1.3 billion tobacco users globally, 80% of whom were from low- and middle-income countries such as Peru^1,20,21^. Despite the WHO recommendation on the implementation of the MPOWER strategy to reduce these figures, the implementation of the comprehensive prohibition of TAPS has only been applied in 57 countries and it is still deficient in Peru^1,6^. It should be noted that the WHO also includes anti-tobacco media campaigns as part of the strategy to decrease tobacco consumption, showing to be effective in reducing tobacco consumption and increasing quit attempts in low- and middle-income countries^1^. Nevertheless, anti-tobacco campaigns have become a neglected practice with only 45 countries running a best practice, Peru not being one of them^1^. All these strategies should be prioritized and their practice accelerated to improve public health following the example of other countries. This is especially important in the population of adolescents, who not only present greater vulnerability to the advertising to which they are exposed but also start to consume tobacco at a very early age, thereby increasing the likelihood of suffering from diseases associated with smoking in the future and reducing their life expectancy^1,22^. An example of success in the implementation of TAPS prohibitions is Spain, which began in 2006 and observed a significant initial effect in the reduction of tobacco use with prevalence rates of 4%, achieving a reduction of up to 6% in the 14 years following its execution, even in the absence of campaigns during the period 2016–2018^23^.

### Limitations

Regarding the limitations of the present study, its cross-sectional nature may preclude the possibility of evaluating causality due to the lack of temporality in the measurement of the study variables. As another limitation, it is necessary to mention that the questionnaire does not specify the duration or contents of the advertisement or if it was commercial or posted by an influencer, and thus, it could not be determined if any particular characteristic is associated with a higher probability of consumption. There may also be a memory and social desirability bias on the part of the interviewees, as well as errors in data recording by the interviewer. In addition, due to the lack of information on exposure to tobacco advertising and missing data, the study had different sample sizes for the three types of exposure, which may reflect differentiated exposure patterns and possible selection biases. Furthermore, the gold standard method for determining tobacco use is the chemical detection of nicotine in saliva, urine or blood, and the secondary report by the interviewees may present the possibility of non-differential misclassification of the outcome^24^. Additionally, there is also the possibility of reverse causation; that is, TAPS may not cause an increase in tobacco consumption, but tobacco consumption predisposes to greater exposure to TAPS. Despite these limitations, the use of the GYTS 2019 is justified because it was the most current source of information at the time of the study and allows cross-sectional estimates to be made for each country regarding smoking and factors associated with its use. Therefore, this survey is useful for the investigation of advertising and its influence on the use of conventional cigarettes in Peruvian adolescents. In addition, the GYTS is a widely supported WHO survey that has a globally standardized methodology that builds representative, independent, and cross-sectional samples, allowing comparison of the state of population health over time and with respect to other countries where it is also used^25^.

## CONCLUSIONS

Our results suggest that TAPS in television and online media are significantly associated with higher consumption of conventional cigarettes in Peruvian adolescents aged 13–15 years. It is necessary to implement comprehensive and complete bans on TAPS in Peru in order to prevent the tobacco industry from finding loopholes to continue introducing tobacco advertising through different media. Bans should be more aggressive in television and online media, which have a high probability of access and very frequent use by adolescents. Finally, further studies are needed for more in-depth evaluation of the mechanisms of the associations identified, the impact of different forms of advertising, and interventions at school and home levels to prevent the initiation of tobacco use among the adolescent population.

## Data Availability

This study used data from the Global Youth Tobacco Survey (GYTS). GYTS is supported by the World Health Organization and the US Centers for Disease Control and Prevention. The dataset used in this article is available in the CDC repository at https://www.cdc.gov/tobacco/global/gtss/gtssdata/index.html.

## References

[1] World Health Organization WHO report on the global tobacco epidemic, 2021: Addressing new and emerging products.

[2] Centers for Disease Control and Prevention Smoking & Tobacco Use: Youth and Tobacco Use.

[3] Peru Ministerio de Salud; Pan American Health Organization; World Health Organization; Centers for Disease Control and Prevention Global Youth Tobacco Survey: Fact Sheet. Peru 2014.

[4] Peru Ministerio de Salud; Pan American Health Organization; World Health Organization; Centers for Disease Control and Prevention Global Youth Tobacco Survey: Fact Sheet. Peru 2019.

[5] U.S. Department of Health and Human Services (2014). The health consequences of smoking: 50 years of progress. Α report of the surgeon general.

[6] World Health Organization WHO report on the global tobacco epidemic, 2021. Country profile: Peru; 2021.

[7] Bardach A, Alcaraz A, Roberti J, Ciapponi A, Augustovski F, Pichon-Riviere A (2021). Optimizing tobacco advertising bans in seven latin american countries: Microsimulation modeling of health and financial impact to inform evidence-based policy. Int J Environ Res Public Health.

[8] Lovato C, Watts A, Stead LF (2011). Impact of tobacco advertising and promotion on increasing adolescent smoking behaviours. Cochrane Database Syst Rev.

[9] Plamondon G, Guindon GE, Paraje G (2017). Exposición a la publicidad de tabaco y consumo de tabaco en adolescentes en América del Sur. Salud Publica Mex.

[10] Satpathy N, Jena PK, Epari V (2022). Gender dimensions of youth vulnerability toward access to cigarettes in South-East Asia: Evidence from global youth tobacco survey. Front Public Health.

[11] Global Youth Tobacco Survey Collaborative Group (2014). Global Youth Tobacco Survey (GYTS): Core Questionnaire with Optional Questions.

[12] Centers for Disease Control and Prevention Global Tobacco Surveillance System Data.

[13] Rao JNK, Scott AJ (1984). On Chi-Squared tests for multiway contingency tables with cell proportions estimated from survey data. Ann Stat.

[14] Lienemann BA, Rose SW, Unger JB (2019). Tobacco advertisement liking, vulnerability factors, and tobacco use among young adults. Nicotine Tob Res.

[15] El-Awa FMS, El Naga RA, Labib S, Latif NA (2018). Tobacco advertising, promotion and sponsorship in entertainment media: a phenomenon requiring stronger controls in the Eastern Mediterranean Region. East Mediterr Health J.

[16] Congreso de la República del Perú (2006). Ley No 28705: Ley general para la prevención y control de los riesgos del consumo del tabaco.

[17] Kapoor S, Lal P, Yadav A (2021). Indirect tobacco advertising, promotion and sponsorships in the Indian Premier League 2020: Tobacco industry’s continuous presence in Indian cricket. Indian J Tuberc.

[18] Marion H, Garner W, Estrada A, Moorer C, Acosta-Velazquez I (2020). Online pro-tobacco marketing exposure is associated with dual tobacco product use among underage US students. Am J Health Promot.

[19] Choi K, Rose SW, Zhou Y, Rahman B, Hair E (2020). Exposure to multimedia tobacco marketing and product use among youth: A longitudinal analysis. Nicotine Tob Res.

[20] World Health Organization (2022). Tobacco.

[21] Organización para la Cooperación y el Desarrollo Económicos; Banco de Desarrollo de América Latina; Naciones Unidas; Comisión Europea (2019). Perspectivas económicas de América Latina 2019: Desarrollo en transición.

[22] Bardach AE, Caporale JE, Alcaraz A (2016). Carga de enfermedad por tabaquismo e impacto potencial del incremento de precios de cigarrillos en el Perú. Rev Peru Med Exp Salud Publica.

[23] Córdoba-García R (2020). Catorce años de ley de control del tabaco en España. Situación actual y propuestas. Aten Primaria.

[24] Martín V, Fernández D, Ordóñez C, Molina AJ, Fernández E, de Luís JM (2008). Valoración con tres métodos diferentes de la prevalencia de consumo de tabaco en estudiantes de primer curso de ciencias de la salud de la Universidad de León en 2006. Rev Esp Salud Publica.

[25] Centers for Disease Control and Prevention (2008). Global Youth Tobacco Surveillance, 2000-2007. MMWR Morb Mortal Wkly Rep.

